# Updating the Discontinuity Theory to the Extended Immunity: The *Symmunobiome* Concept

**DOI:** 10.1002/eji.202451528

**Published:** 2025-04-19

**Authors:** Federico Boem, Ingrid Lamminpää, Amedeo Amedei

**Affiliations:** ^1^ Dipartimento di Scienze Politiche Giuridiche, Sociologiche e Umanistiche Università degli Studi “Niccolò Cusano Rome Italy; ^2^ Department of Clinical and Experimental Medicine University of Florence Florence Italy; ^3^ Network of Immunity in Infection, Malignancy and Autoimmunity (NIIMA) Universal Scientific Education and Research Network (USERN) Florence Italy

## Abstract

The immune system (IS) is commonly understood as a system composed of specific cells and tissues that have evolved to contrast pathogens and defend the host. By virtue of this capacity, it has come to be considered capable of making an essential distinction, that between self versus non‐self, which would contribute to a clear identity of the organism. However, in the wake of evolution and ecology, growing evidence suggests that the so‐called immune system, which also evolved from symbiotic interactions with external agents, is not just a defensive system that merely protects the organism but, on the contrary, is involved in many global regulatory and homeostatic functions. Moreover, in performing these many functions, IS is not only an ensemble of host cells and tissues but functionally is constitutively determined by the interaction with a set of associated microorganisms, that is, the human microbiome. In this scenario, it is open‐and‐shut that the microbiome itself is a functional part of this extended immune system. Organisms and microbiomes together, therefore, form a functional whole, which constitutes a privileged form of biological organization. In light of this evidence showing the inadequacy of traditional accounts, we propose to extend and supplement the current IS conceptualization by introducing the notion of the symmunobiome. With this term, we intend to characterize the microbiome's own and unavoidable component to overall immune functionality. Therefore, we suggest a new immune system determination, articulated in three linked pillars—adaptive immunity, innate immunity, and symmunobiome—to better grasp the diverse functionality of extended immunity.

## Graphical abstract

The evolution of the concept of the immune system from a set of composed cells and tissue that defend the host from exogenous agents into a system that has regulatory and homeostatic functions determined by the interaction with microorganisms as a functional part of an extended immune system, that we call symmunobiome.

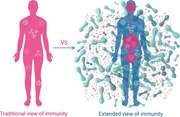

## 1 Introduction

Immunology is generally understood as the biomedicine branch that deals with studying the mechanisms and structures that an organism displays to protect itself against threats (both external and internal) from agents or factors that undermine its material and functional organization. Immunology has a pervasive character as its topics of investigation intersect with other different fields of research. Obviously, this concerns infectious diseases, but also, most outstandingly, cancers, allergies, and autoimmune and metabolic diseases [1]. As a matter of fact, an immunological perspective can be virtually found and expected in many areas of biomedical investigation, since immunity is a diffuse (within the whole organism) and dynamic system (in the sense also of its plasticity). The systemic aspect of the immune system (IS) is due to the fact that, throughout evolution, it involves the coordinated activities of many specialized cell types that interplay within cells and tissues of the entire organism, allowing it to respond, more or less, specifically to the plethora of stimuli and stresses to which the organism is subjected [1]. Traditionally, the immune system has been divided between innate and adaptive ones. Evolutionarily speaking, the former is the most ancient one (present in both vertebrates and invertebrates). The innate immune system is labeled as such since so‐called innate cells (e.g., NK cells, macrophages) express receptors recognizing macro classes of conserved antigens, thus providing a fast response to, for instance, viruses and bacteria. Conversely, B and T lymphocytes, classes of immune cells, are said to belong to the adaptive system since they are capable of developing a more specific and tailored immune response due to the recognition of specific antigens through their B cell receptor (BCR) and T cell receptor (TCR), respectively. These receptor genes undergo processes such as V(D)J recombination (through the activity of recombination‐activating genes or RAGs), somatic hypermutation, and affinity maturation, which allow their diversification at the level of somatic differentiation. In fact, the lymphocytes have a different genome with respect to the other organism's cells [2]. Although this way of looking at the immune system is definitely crucial and useful, the evolutionary IS trajectory within life history is obviously not linear, and the reconstruction of such a complex scenario is still ongoing (including the determination of the role of symbiosis). The general idea in the end is that the immune system evolved, in its various functions, in relation to and in response to host relationships with other organisms (e.g., so‐called pathogens) and primarily to protect ourselves against them.

**Summary**

• In this paper, we attempt to challenge the traditional separations between immunological functions (innate and adaptive) by placing them within a common framework, borrowed from the discontinuity theory of immunity, including the microbiome, which thus becomes an actual component of an extended account of the immune system.

• We therefore also propose a new terminology and indicate some future steps for the experimental implementation

## 2 The Immune System Beyond Defense

Although these approaches and classifications remain fundamental, they are now seen as more reductive [3, 4, 5]. While it is true that the functions of the organism (including primarily immunity itself) have evolved in relation to other living forms that interacted profoundly with us, these relationships cannot be reduced to mere pathogenicity. Moreover, this dynamic continuum is profoundly context‐dependent and determined by evolutionary and ecological aspects, which show that pathogenicity cannot be understood as an intrinsic property but rather should be intended as a relational, and thus non‐static, one [1, 6, 7]. Moreover, an increasing number of studies have shown that the IS activities go beyond the organism's defense [8, 9]. Mounting evidence suggests that IS is involved in the establishment and regulation of many different organismic phenomena. Indeed, immunity plays a crucial role in the determination and maturation of many anatomo‐physiological structures during development, it is fundamental for the repair and functional preservation of diverse tissues, it modulates more metabolic functions, and it is involved in various other activities that make it a kind of master regulator of the organism's overall homeostasis [5]. Although certainly crucial and reflecting the historical development of immunology, focusing exclusively on the defensive response also fails to account for the IS many more complex relationships, both internal and with the external environment and other organisms. In the following sections, we aim to show how, in our opinion, some core concepts of traditionally understood immunity should be updated in the light of new evidence and the innovative theoretical framework that sees the IS as part of a symbiotic relationship.

### 2.1 From “self vs. non‐self” to the “liquid self”

Given its global role in organismic functionality, the immune system is often regarded as a constitutive element of organismic identity [10]. As already stated, the traditional hypothesis is that in the evolutionary trajectory, a defense system of the organism emerges to preserve its internal coherence from disruptive changes.

As already said, this conceptual and terminological stance definitely mirrors the historical development of immunology as a research field: the immune system was initially studied in relation to its function against external infections, explaining that the main theoretical distinction of contemporary immunology hinges on “the self vs. non‐self” concept [10]. Following this perspective, the organism's own identity would constitute the *self* because, under physiological conditions, it does not trigger the immune response, whereas everything outside the organismic identity is the non‐self since it instead does it. Accordingly, immunity would provide a criterion for biological identity since such an identity relies on distinguishing the self from the non‐self [1].

Although well established as a theoretical framework, the distinction between self and non‐self presents several challenges for a general understanding of immunological mechanisms. The phenomena belonging to self‐reactivity show how the discrimination between self and non‐self is much more nuanced than we think, suggesting a broad spectrum of situations ranging from mild activity to autoimmune diseases [6, 11, 12, 13]. Moreover, as a matter of fact, IS reacts quite differently (along a spectrum ranging from mild, modulating interactions to aggressive responses) to numerous elements that, according to the traditional view, would not represent the self: first and foremost, the so‐called microbiome (i.e., the collection of all the microbial populations residing in and on a host, including bacteria, fungi and viruses). This spectrum of situations shows how different contexts can make the categorization of antigens more blurred, making them fluctuate between self and non‐self and thus promoting the idea of a fluid or liquid self [1, 10].

### 2.2 Discontinuity Theory

Because of the issues and difficulties of the classical theoretical framework, which proposes a too stiff and discrete distinction between self and non‐self, and yet not to renounce the idea of a unifying theoretical perspective, recently it has been proposed that the immunity should be seen as a system aimed to recognize discontinuity [4].

According to the “discontinuity theory”, the immune system is able to detect and respond to changes in the antigenic exposure (to the rate and the intensity of the “signal” and not just to antigens as such) and, at the same time, it can be able to adapt to it, becoming “tolerant”, if the stimulus turns into continuous (chronic) [4]. In other words, the discontinuity theory argues that the immune system registers and reacts to sudden changes in antigenic stimulation. Thus, within this theoretical framework, the human immune system would have evolved (also in relation to our symbionts) to adjust its activity to rapid changes in antigenic stimulation and then become tolerant to slow or continuous stimulation. For instance, in the case of a classic infection, the immune system would detect antigenic changes over time, thus becoming receptive to sudden changes, providing an adequate explanation for the activation of effective and targeted immune responses. Conversely, when antigenic concentrations were high but continuous and characterized by low levels of variation in antigenic concentration, this would lead to so‐called immune tolerance. Summing up, this means that chronic and constant exposure to antigens would lead to immune tolerance, whereas sudden antigenic changes would provoke specific immune responses. In addition, this would also explain how persistent exposure to constant immune stimuli can reduce the response of immune cells (both adaptive and innate) to a signal that becomes “part” of the system [14]. The discontinuity theory thus provides a unique and comprehensive theoretical framework according to which all the different modes of immune response (often considered distinct and independent phenomena) can now be traced back to a kind of general mechanism that sees the immune system as a sort of pattern recognition system. From a more abstract point of view, and following Luciano Floridi's [15] diaphoric interpretation of data, we can define data as “lack of uniformity”, thus as anything that can be recognized, detected, or quantified as different from the background state (thus capturing not only the presence of a new signal and its magnitude but also the absence of a well‐established previous data). If we look at discontinuity theory with this idea of data in mind, it becomes clear that IS can be seen as a sort of informational system of the organism, meaning a dynamic structure able to detect variations within a uniformity and to give it a meaningful role in relation to the activity of the system and its response.

Because of that, we argue that the discontinuity theory has shown to be a plausible account for different scenarios, including infections led by viruses, tumor onset and progression, and allergic reactions. In the first two cases, experimental evidence clearly shows that after a first reaction, chronic exposure eventually leads to loss of the immunity function, suggesting that prolonged contact may shape a new “continuity,” thus no longer detectable. Since either the variation or reoccurrence of the stimuli on a given background is the key for detection, this may explain why chronic inflammation (such as in allergies) does not induce immune tolerance [4].

Historically, immunology has treated distinctively the variety of mechanisms underlying IS activities (such as pattern and nonpattern recognition, tissue damage, and changes in functional activity). Indeed, all these cases have peculiarities that, experimentally, deserve to be evaluated in their context of action. However, this does not abolish the theoretical possibility that all these cases might be instances of a common activity (shaped by evolution), that sees the identification and response to changes as the essential characteristic traits of the immune system.

### 2.3 Extending discontinuity

In its original formulation, the discontinuity theory refers to the immune system as limited to just specific cell lineages (i.e., leukocytes). Here, we envisage the need to extend it to the microbiota. The microbiota generally refers to communities of microorganisms (such as bacteria, fungi, and viruses) that inhabit certain places in the human body and form a symbiotic relationship with it from both a physiological and evolutionary point of view [5, 12, 16]. The microbiome, on the other hand, refers more frequently to the genomes of all these associated organisms and their ecological relationships. However, the literature is not always consistent about this distinction and it is often easy to find “microbiome” and “microbiota” as synonyms. For our work, we refer here more generally to the microbiome, as it has a broader scope. Moreover, historically, the most studied and relevant microbiome has been the intestinal microbiome, but recent studies show that other areas (niches) of the human body (such as the skin, mouth, or respiratory system) are populated by specific microbiomes and that these interact both locally with specific tissues and more globally, first and foremost with the IS [17]. By virtue of the pervasive and predominant role of the microbiome for various physiological functions, and given its very co‐dependence on the functions of the immune system, we propose here that it could be considered a proper functional IS component [5]. By this, we do not only mean to refer to the already well‐established and abundant body of evidence concerning the microbiome's role in the activities of the IS but to argue that the microbiome itself contributes, constitutively, to the boundaries of the immune system's own proper range of action. This implies the idea of extended immunity that therefore belongs to a functional whole that goes beyond the human organism and instead includes the human body and its associated microorganisms: that is, the so‐called holobiont [14, 16, 19, 18]. To do this, we will now detail the main aspects and mechanisms according to which the interaction of the microbiome with immune functions is not only supportive but also fundamental. This will allow us to specify what we mean by ‘functional part’ and why, according to this understanding, the microbiome can be seen as a functional pillar of the immune system. So, from this perspective, this also means that the microbiome is to be considered as a constituent element and therefore generates a constant but dynamic immune signal. The quality and any differences in response to this signal can obviously concern both the intrinsic variations in the microbiome composition (which, as we know, can undergo modifications due to both exogenous factors, such as diet, and endogenous factors such as genetic level) and the impact that the microbiome has on the modulation of the other components of the extended immune system, such as leukocytes but also other cell types, such as intestinal epithelial cells. However, this process can also occur in the opposite direction, that is, the microbiome can be modulated by the activities of the human component of the holobiont. If we accept this proposal and therefore consider the functional holobiont unit, it appears clear that the microbiome itself becomes a legitimate functional extension of the discontinuity recognition system, both by direct and indirect activity (see also section 4). In emphasizing these aspects our proposal is similar to that put forward by Eberl [3] but differs from it in some respects. Indeed, we believe that the concept of holobiont is more suited to our proposal than that of superorganism, since while the latter refers to the association of individual and conspecific organisms in forms of collective organization (such as colonies of eusocial insects), the holobiont concept specifically refers to the creation of functional units of biological organization that are composed of different living entities, even belonging to different species and other taxonomic categories [16].

## 3 Immunity: A Combined Mechanism of Action

In accordance with this perspective, we believe that the one between the immune system and the microbiome is not only so‐called crosstalk but that understanding the activities of the functional whole is not fully accessible without recourse to the notion of the holobiont (understood precisely as a unit of biological organization). If one accepts this point of view, it becomes clear that one is faced with a reversal of the classical perspective. That is to say, it is not the concept of extended immunity that is an abstraction or construction based on the interactions between the IS and the microbiome, but instead, it is these latter that are abstractions and reductions, useful in certain cases, but which risk failing to account for this broader level of biological organization and from which many physiological functions such as those found in immune activity are derived (we will come back to this point in the further section). Indeed, these functions are not just local but show a global range since the effects of this extended immune functionality have been documented at both local and distal sites. For instance, germ‐free mice are found to have many immunologic deficits, including incorrect differentiation of T cells, decreased secretion of immunoglobulin (Ig) A and production of antimicrobial peptide, and systemic IgE increase [20]. In humans, a gut microbiome depletion and consequent reduction of its peculiar by‐product short chain fatty acids (SCFA) brings to a condition referred to as “leaky gut” which is common to several inflammatory disorders such as obesity, cardiovascular disease, cancer, but also to neurological conditions (amyotrophic lateral sclerosis [ALS], Alzheimer's and Parkinson's diseases), immune disorders (inflammatory bowel disease [IBD], multiple sclerosis) and behavioral disorders (e.g., autism) [21].

Indirectly, diet patterns and lifestyle play a pivotal role in chronic inflammatory, cancer [22], autoimmune, and neurodegenerative diseases [23] by shaping the microbiome composition. For instance, whole plant foods are the main source of dietary fibers which are converted into SCFA in the distal part of the intestine. Processed and refined food is fermented in the small intestine, leading to bacterial overgrowth and a microbial signature that negatively impacts the immune response. In humans, immune priming begins in utero. Maternal microbiome and a diet rich in fibers start shaping perinatal immune factors such as cord‐blood IgA, immune cells, and cytokines. From birth, breast milk‐associated Bifidobacterium is directly related to IS programming and maturation: it guides gut epithelial barrier development and increases levels of the regulatory T‐cells (Tregs), circulating interleukin (IL) 10, and anti‐inflammatory monocytes.

The microbiome is further implicated in promoting angiogenesis and epithelial cell development in the intestinal barrier. That explains why at birth, humans have a relatively underdeveloped immunity and a tolerogenic milieu to simplify the cohabitation of microorganisms with the host without promoting inflammatory responses. A proper example is represented by segmented filamentous bacteria (SFB), sporogenous Gram+ anaerobic bacteria that promote the differentiation of Th (T helper)17, Th1 and stimulate IgA production in the gut and are among few commensals able to firmly attach to Peyer's patches and epithelial cells by inducing the reorganization of their cytoskeleton at the contact point. Within the homeostatic condition, SFB are used by the host to strengthen tissue immunity and responses to other exogenous microorganisms. An additional example is given by commensal bacteria, which express flagellin, a structural protein that interacts with Toll‐like receptors in case of intestinal barrier disruption. Dendritic cells (DCs) residing in the lamina propria respond to flagellin by secreting antimicrobial peptides and cytokines (such as IL‐23), which induce innate lymphoid cells to release IL‐22, leading to epithelial protection [24].

More generally, the microbiome is actively orchestrating immune response with other host‐dependent immune cells and factors: (1) competing with pathogens for the same nutritional substrates (colonization resistance); (2) making the environmental niche unfavorable to other newcomers by altering PH; (3) secreting antimicrobial peptides; (4) using metabolites (e.g., SCFAs) which are able to modulate the immune response through down‐ or upregulation of gene expressions. Healthy microbiome (a definition that is not immune to difficulties and still not entirely clear, but which nonetheless, given its association with the functionality of healthy host organs and tissues, highlights its systemic‐symbiotic nature [25]) plays a great effort to reinforce the intestinal epithelial barrier (IEB) to contain and protect their ecological microenvironment. IEB function, made by mucus, IgA, immune cells, and antimicrobial peptides, is to minimize contact between the epithelial cell surface and bacterial or exogenous factors in the lumen, and its health is granted by SCFAs’ abundance. Among SCFAs, butyrate is able to induce the IL‐10 secretion and Tregs’ expansion, inhibit the histone deacetylase 3, and further stimulate monocyte differentiation to macrophages [26]. In addition, butyrate has been seen to increase MUC gene expression for mucin, a component of the mucosal layer [27]; the same result in mice has been achieved by certain phages [28].

In a cohort of ALS patients and healthy controls (HC), specific circulating SCFA are consistently lower in ALS patients compared with HC. A major role is also played by blood virome and, in detail, the load of Torque Teno virus, higher in ALS patients than in HC, becomes a valuable biomarker, predictive of the disease progression [29]. Therefore, it is needed to extend to the virome as well, the ability to elicit an immune response, having viruses exerted and still exerting a critical role in the coevolution of physiological processes of our human species [30].

SCFAs are also seen to have an impact on DCs production of retinoic acid, which is affecting Th17 and Tregs populations. When epithelial cells are activated, they also activate DCs and macrophages by enhancing their ability to present antigens to T cells residing in the gut intraepithelial and the lamina propria. Additionally, they can facilitate DC migration to peripheral lymph nodes, mesenteric lymph nodes, and Peyer's patches, where they can stimulate naive T cells starting immune responses to microbial antigens at distal locations [24].

Among SCFAs, propionate is also able to induce Tregs secretion of IL‐10 that consequently leads to Th17 cells inhibition. In patients with end‐stage renal disease, propionate enhances the concentration of Tregs and C‐reactive protein in peripheral blood. In addition, a recently developed bacterial consortium “GUT108” has been tested positively on the decrease of CD4+T cells and several inflammatory cytokines [31]. Finally, lactocepin, a by‐product of *Lactocaseibacillus casei*, is involved in IP‐10 suppression and consequently in decreasing inflammatory processes, resulting to be therapeutic for IBD patients [32].

Given these results, it is evident that by changing the cytokines’ level, the gut microbiome exerts an effective influence on the migration of immune cells. In tumors, immune checkpoint therapy (ICIs) efficacy is likely dependent on the composition of the gut‐microbiome: in detail, it is directly linked to an abundance of *Akkermansia muciniphila* [33]. In addition, the *Bifidobacterium fragilis* and *Bacteroides thetaiotaomicron* play a key role in the effectiveness of CTLA‐4 inhibition on cancer cells. IL‐12‐dependent Th1 immune responses are influenced by the microbiome composition, and these responses help reduce tumors in murine models and human trials while maintaining intestinal integrity [34]. Moreover, gut‐derived *Streptococcus* may migrate in the bloodstream, colonize the intratumoral tissue, modulate the immune infiltration within the tumor microenvironment, and increase the immunotherapy efficacy [22].

It is relevant, though, to remember that quite often, the interaction with the microbiome and the ICIs is not a matter of a single species but more of a cohort‐specific microbial signature.

Conversely, microbiome depletion in intestinal tumors, together with a thin mucus layer, brings bacterial translocation, increasing concentration of Th17 cells, and a consequent hyperimmune response against dysregulated commensals. A study conducted on colorectal cancer patients, by Amedei et al. [35] highlights how different strains are colonizing tumor and healthy tissue in the same patient. Prevotella bacteria are prevalent in cancer mucosa samples and Bacteroides in surrounding healthy mucosa samples, and they have, respectively, a positive and negative correlation to IL‐9. These results corroborate the idea of the existence of bidirectional interactions between the immunity and the host commensal bacteria.

## 4 Extending the Discontinuity Theory: The *Symmunobiome and Symmunobiont*

As shown, the reciprocal influence of the microbiome on the immune system is now well documented and established. However, we have already mentioned that these studies, although fundamental, are still embedded in a paradigm that sees the microbiome and the IS as strictly separate systems.

Indeed, we believe that further specification can be made in this scenario, which is also the sense of our theoretical proposal. It is precisely through the notion of the holobiont that it is possible to appreciate the constant interaction (we could say almost “dialectical”) [36] between these two components of the extended IS (i.e., the immune cells and the microbiome) opens up new perspectives, reaffirming the context relevance in determining, for instance, the pathogenicity of a microorganism, which becomes a contextual property, dependent on genetic, spatial and ecological (thus overall systemic) factors [37]. This, therefore, means that the perspective according to which the immune system of the host organism and the microbiome influence each other as quite rigidly distinct entities can be partially revised.

Accordingly, the immune system no longer acts just as a barrier, but rather as a fine‐tuned functional modulator of a greater interaction, where mechanisms (largely to be discovered) govern the determination of some elements as self and others as non‐self, thus confirming the liquid and contextual nature of this distinction [10]. This is from both a physiological and evolutionary perspective [5, 12]. The boundary between the immune system and the microbiome is, therefore, not an impermeable wall but a porous belt with reciprocal exchanges and mutual influences. Moreover, this does not only occur in specific, localized tissue‐related areas but affects the organism as a whole. Thus, on the one hand, microorganisms play a decisive role in determining the functional IS conformation. On the other hand, the immune system itself is responsible for the ecological balance and variety of activity of the symbiotic populations of these microorganisms. Accordingly, relations among organisms are far more complex than previously determined and the immune system is much more than what its name suggests. As already explained, holobionts challenge the traditional “individual boundaries” of those entities we call “organisms”. If living beings previously classified into animal and plant species are no longer uniform individuals, they can now be seen as systems of interactions and functional assemblages of different components, that is, the host and its heterogeneous associated microorganisms or the holobiont. Since holobionts are suggested to constitute a new level of biological organization (and therefore worthy of specific investigations), (even as units of evolution) they could also legitimately be considered as potential new targets of specific therapeutic approaches, with an eye toward personalized medicine [16].

Practically speaking, within the holobiont perspective, we envisage the need to consider the microbiome itself as a legitimate part of this (extended) immunity. Fluctuations in the host‐dependent immune response have long been studied and would have a correspondence with microbial ones given the dual valence that the same microorganisms assume on the basis of different concentrations and locations. The definition of immune response, in light of the interactions reported so far, can hardly be comprehended through solely the mechanisms of action of innate and specific immunity (and focusing only on so‐called immune cells), but necessarily requires an extension that complements the first two or even, anticipates them, in follow up to the superorganism concept. Thus, in this functional perspective, IS is not a defender, and even in its extended regulatory and homeostatic functions, it does not pertain solely to the host but to a system that emerges within the holobiont and precisely regulates its boundaries and identity [3]. In light of these observations, we have shown that some authors have already proposed different accounts for this conceptual change, focusing both on redefining the fuzzy nature of the opposition self versus non‐self (thus rather suggesting its liquid characterization [10]), and on conceiving the IS interaction with the microbiome as generating an extended functional unit (i.e., the holobiont).

Because of that, in line with these previous proposals and by broadening the traditional concept of immune functionality, we believe that is the appropriate time to introduce a new lexicon reflecting this conceptual shift in a way that takes into account and updates previous attempts within a common framework. We therefore hope that in this, those working on conceptual alternatives who want to do justice to the new evidence can find themselves in our proposal. Therefore, on the one hand, we would like to introduce the concept of the symmunobiome to characterize the microbiome's contribution to the functionality of the immune system as extended. By this, we mean that part of the functional activities traditionally ascribed to the classical IS alone is instead performed (more or less autonomously) by the microbiome as such. In other words, such a notion would constitute the immune component represented by the microbiome itself. This implies, in turn, the existence of an immunobiont, that is, a functional immune whole. By this term, we mean the immunological counterpart of the holobiont/superorganism concept. This means that to speak of an immune system would be to consider only a part of the individual's immune functionality, which is instead determined globally by the symbiotic relationship. As with that of the holobiont, we believe that, in view of both scientific and clinical practice, the immunobiont concept is not simply a semantic innovation but concerns a different way of studying specific issues such as specific inflammatory responses or autoimmune diseases. In this sense, inspired by the work of Bordenstein and Theis [16], we think it is appropriate to specify some fundamental points. Being the immunological specification of the holobiont, the immunobiont has to be understood as a unit of biological organization, and the systems comprising it (immune system, microbiome, cellular activities of specific tissues) can only be considered distinct as an abstraction. In other words, what we usually have ascribed to the immune system is indeed a result of the activities of the immunobiont. Moreover, the functions of the immunobiont are such (and can only be understood) by virtue of their symbiotic nature. It follows that a more profound and comprehensive insight into immune functions occurs if we consider the parts of this symbiotic relationship together; thus, neglecting any of them could lead to distorted or deficient conclusions.

Accordingly, we propose to incorporate this new conceptualization within the traditional classification, effectively updating and extending it. We suggest a novel IS structure in three linked pillars: adaptive immunity, innate immunity, and symmunobiome to better grasp the diverse functionality of the extended IS, thus the symmunobiont. From this perspective, given that the discontinuity theory has been presented as a characteristic of the immune system, by extending this system to a higher level of biological organization, we believe that the microbiome, along with the traditional immune system and the other types of cells (i.e. those involved in the complex interactions and signals of the extended immune response) more properly intercept the possibilities of monitoring and responding to signal changes. Accordingly, we want to argue that the symmunobiome as such should be the functional core to be considered in the discontinuity theory. As already stated, this innovation should not be seen as just theoretical. If a new class of objects and new forms of relationships are established, it is also required to specify methods and procedures to study their properties. We also believe that such theoretical definitions, by incorporating the development of new protocols that consider microbial population dynamics and its associated metabolomics, customized to the individual, would have an impact on future innovations both in research methods and in efforts to understand an individual's immune response. For instance, diet and lifestyle directly affect the microbiome composition and gut‐associated immune cell repertoire by improving the efficacy of cancer immunotherapy treatments and enhancing antitumor immunity or by suppressing inflammation and ameliorating autoimmune diseases like type 1 diabetes, multiple sclerosis, and inflammatory bowel disease [38, 39]. Besides diet and lifestyle, microbiome‐based therapy such as prebiotics and probiotics administrations, or fecal microbiota transplantation (FMT), and even possibly helminthic therapy [37] could become, in the light of a greater symmunobiome understanding, an approach within immune therapies, which would also be moved toward the idea of extended immunity. Indeed, since these approaches represent already feasible strategies to select host‐beneficial strains, under this new proposal they could provide a more adequate understanding of the functional dynamics of the host–microbiome interactions. In addition to that, imaging methods to monitor real‐time population abundance, computer simulations that forecast the dynamics of complex species, and the ways in which nutrition and/or substances released or displayed on intestinal epithelial cells affect the microbiome will be the subject of future research. Comprehending the precise mechanisms by which the microbiome influences cellular physiology, the immune system in both proximal and distal organs, and neurophysiology is critical.

## 5 Conclusion and Future Perspective

From the given variety of examples explored in this paper, it is evident how the microbiome can be seen as a functional pillar of immunity whose functional contribution we refer to as symmunobiome. By this term, we mean the functional immune unit constituted by the human and the microbiome immune components. This also means that the formal structure of the discontinuity theory (which holds that the immune system has evolved, symbiotically, as a system capable of detecting and reacting to changes in antigenic exposure) should be extended to this functional unit. In this regard, being the distinction between host‐dependent (innate and adaptive) and host‐independent immune response no longer sustainable as such, standardized research protocols should be revised in light of this new immunity concept, including microbiome characterization and its by‐product evaluation to better assess health status or determine disease onset, prognosis, and progression. Moreover, besides diagnostic protocols, guidelines on diet and lifestyle, which can directly affect the microbiome composition and gut‐associated immune cell repertoire, should be implemented. In addition, the so‐called microbiome‐based therapy (MBT) in both accounts as a biomarker of pathological condition and as a modulator (through prebiotics and probiotics administrations or FMT), could be seen within the repertoire of (extended) immune therapeutic interventions. Nevertheless, MBT still needs to be better integrated, in terms of experimental procedures and clinical protocols, within this expanded framework, to better fulfill the promise of personalized healthcare. To do so, a better understanding of the dynamics of the symmunobiome within the extended immunity should be, in our view, a primary investigation aim in this field. Accordingly, imaging methods to monitor real‐time population abundance, computer simulations that forecast the dynamics of complex species, and the ways in which nutrition and/or substances affect GM will be the subject of future research. Given the difficulties of the traditional accounts (still reflected in the textbooks on which future researchers still study) in providing an adequate explanation and conceptualization of the growing evidence about extended immunity, we hope that our proposal will stimulate a discussion within the scientific community, narrowing the gap between different areas of investigation (such as immunology and microbial ecology) and helping to steer research toward new, more integrated and multidisciplinary approaches.

## Author Contributions

Federico Boem, Ingrid Lamminpää, and Amedeo Amedei: Conceptualization, writing original draft, and methodology. Amedeo Amedei: Final editing and supervision.

## Conflicts of Interest

The authors declare no conflicts of interest.

## Data Availability

Data sharing is not applicable to this article as no datasets were generated or analyzed during the current study.
